# Policy: Chrysotile on Ice

**DOI:** 10.1289/ehp.115-a130b

**Published:** 2007-03

**Authors:** Rebecca Renner

Parties to the Rotterdam Convention, a group of more than 100 countries that have agreed to share information about hazardous chemicals, elected in October 2006 not to add chrysotile asbestos to the list of hazardous chemicals subject to right-to-know export controls. The 1998 Rotterdam Convention currently requires prior informed consent (PIC) for more than 30 chemicals, meaning an exporting nation must ensure that the substances do not leave its territory without the informed consent of recipient countries.

This is the second time the Convention has declined to list chrysotile, a track record that raises serious concerns about the future of the agreement, according to Carl Smith, vice president of the nonprofit Foundation for Advancements in Science and Education. “Listing isn’t a ban,” he says. “It’s just an agreement to share information.” He adds, “Chrysotile would be on the PIC list already if the member countries would just follow through on the agreement they made when they joined the convention. If parties are going to start ignoring the Convention text, the train is off the tracks.”

Chrysotile fulfills all the requirements for listing, Smith says, but unlike many of the other chemicals on the list—such as polychlorinated biphenyls, lindane, and all other types of asbestos—chrysotile is still economically important. Use of asbestos in Western countries has declined due to health concerns, but chrysotile-based products such as pipes and roof shingles are still widely used in the developing world. Trade in chrysotile is worth $600 million a year, according to a 7 November 2005 *Wall Street Journal* estimate.

Prior to the October meeting, a panel of 31 experts on the convention’s Chemical Review Committee determined that chrysotile met the criteria for listing. Most of the meeting participants supported the proposal to list chrysotile. But Canada, Ukraine, Russia, Kyrgyzstan, India, Iran, and Peru objected and blocked action, citing either scientific uncertainty about chrysotile’s health effects or the material’s usefulness.

The Chrysotile Institute, a nonprofit organization funded by the Canadian government, maintains that chrysotile is not as toxic as amphibole asbestos. Institute president Clément Godbout says the high rates of respiratory disease and cancer associated with asbestos stem from exposure to the amphibole form and to high exposures from dangerous past practices such as blowing asbestos mixtures onto walls for insulation and fireproofing. Used properly, chrysotile is a cost-effective ingredient for the cement that is often used in water pipes in underdeveloped countries, he contends.

But many groups say that the concept of controlled use, particularly in developing countries, is a fallacy. Chrysotile is classified as a human carcinogen by the WHO, the Collegium Ramazzini, the World Trade Organization, and other groups. In the meantime, the parties to the Convention have deferred further consideration of the issue until their next meeting in 2008.

## Figures and Tables

**Figure:**
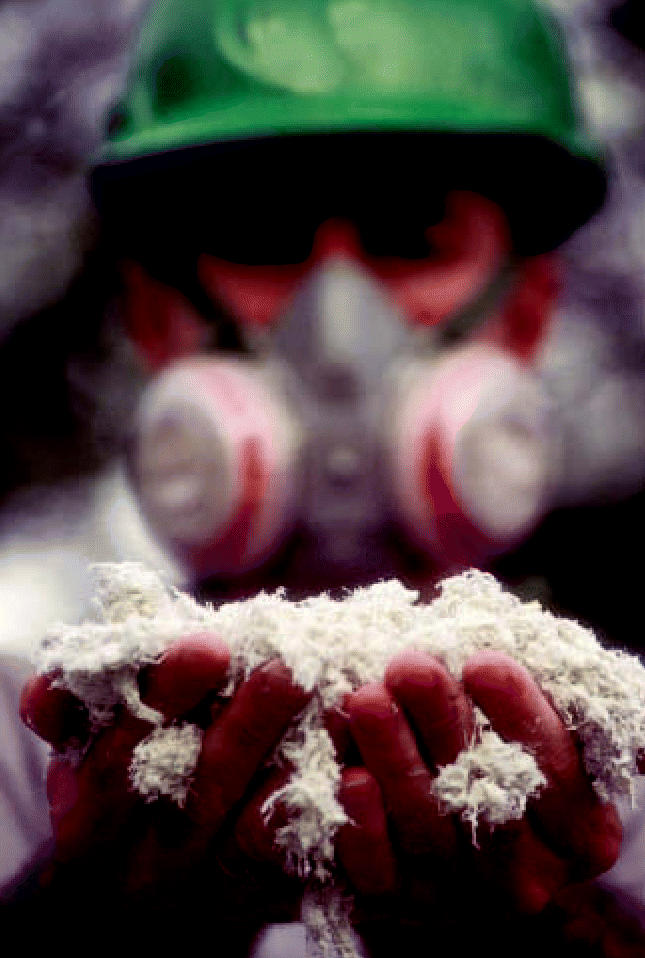
The stuff of controversy Chrysotile asbestos was not added to the Rotterdam Convention despite concerns about its health effects.

